# Current/Voltage Controlled Quadrature Sinusoidal Oscillators for Phase Sensitive Detection Using Commercially Available IC

**DOI:** 10.3390/s20051319

**Published:** 2020-02-28

**Authors:** Winai Jaikla, Suchin Adhan, Peerawut Suwanjan, Montree Kumngern

**Affiliations:** 1 Department of Engineering Education, Faculty of Industrial Education and Technology, King Mongkut’s Institute of Technology Ladkrabang, Bangkok 10520, Thailand; suchin.ad@kmitl.ac.th (S.A.); peerawut.su@kmitl.ac.th (P.S.); 2 Department of Telecommunications Engineering, Faculty of Engineering, King Mongkut’s Institute of Technology Ladkrabang, Bangkok 10520, Thailand

**Keywords:** phase sensitive detection, voltage controlled oscillator, quadrature sinusoidal signal, impedance spectroscopy, commercially available, LT1228

## Abstract

This paper presents the quadrature sinusoidal oscillators for a phase sensitive detection (PSD) system. The proposed oscillators are design by using the commercially available ICs (LT1228). The core oscillator consists of three LT1228s: two grounded capacitors and one resistor. By adding four resistors without the requirement of additional active devices, the amplitudes of two quadrature waveforms become adjustable. The quadrature output nodes are of low impedance, which can be connected to the impedance sensor or other circuits in a phase sensitive detection system without the need of buffer devices. The amplitudes of the quadrature waveform are equal during the frequency of oscillation (FO) tuning. The frequency of oscillation is electronically and linearly controlled by bias current or voltage without affecting the condition of oscillation (CO). Furthermore, the condition of oscillation is electronically controlled without affecting the frequency of oscillation. The performances of the proposed oscillators are experimentally tested with ±5 voltage power supplies. The frequency of the proposed sinusoidal oscillator can be tuned from 8.21 kHz to 1117.51 kHz. The relative frequency error is lower than 3.12% and the relative phase error is lower than 2.96%. The total harmonic distortion is lower than −38 dB (1.259%). The voltage gain of the quadrature waveforms can be tuned from 1.97 to 15.92. The measurement results demonstrate that the proposed oscillators work in a wide frequency range and it is a suitable choice for an instrument-off-the-shelf device

## 1. Introduction

**Figure 1:**
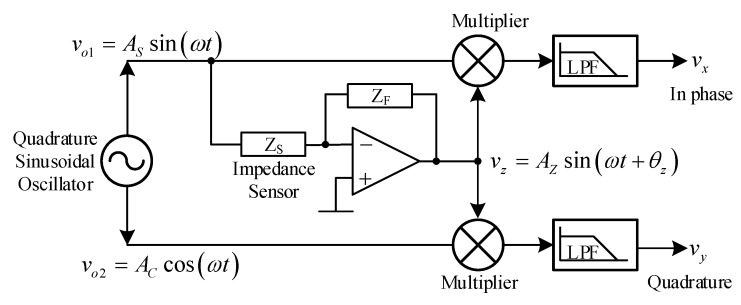
Basic block diagram of the phase sensitive detection (PSD) system.

The quadrature sinusoidal oscillator is the most significant part in phase sensitive detection (PSD), which is an important function in instrumentation and measurement systems. Generally, the electrical signal generated from the sensors include the noises which come from many sources, for example, power systems, pre-amplifiers, thermal noises, etc. Phase sensitive detection is used to detect and measure a very low level electrical signal from the sensor [1,2,3]. The principle of the dual phase sensitive detection system is shown in Figure 1. The quadrature sinusoidal oscillator generates the pure sinusoidal waves, v_o1_ (sine wave) and v_o2_ (cosine wave) with a 90 degree phase difference. The sine wave v_o1_ is fed to the impedance sensor Z_S_, which is connected to an auto-balancing bridge circuit consisting of an op-amp and a feedback resistor Z_F_ [1]. The auto-balancing bridge circuit provides the output $v_{z}=A_{Z}\sin\left(\omega t+\theta_{z}\right)$, where A_z_ and $\theta_{z}$ are the amplitude and phase related to the resistance and reactance components of impedance sensor Z_s_. The signal v_z_ is sent to multiply with the sine wave v_o1_ and cosine wave v_o2_. Then, the DC component is taken out by the lowpass filter (LPF) circuit, following v_x_ and v_y_ [1]:

(1) $$v_{x}=A_{S}A_{Z}\cos\left(\theta_{z}\right),$$

and

(2) $$v_{y}=A_{C}A_{Z}\sin\left(\theta_{z}\right).$$

Equations (1) and (2) show that v_x_ and v_y_ are the real and imaginary components of the impedance sensor [1]. 

The quadrature sinusoidal oscillator can be designed to achieve the electronic controllability for modern control systems. With this feature, the parameters of the oscillator, such as the frequency of oscillation (FO) and the condition of oscillation (CO), can be easily controlled by a microcomputer or microcontroller. This sort of oscillator is called a voltage controlled oscillator (VCO) or current controlled oscillator (CCO). Many voltage controlled oscillators are found in sensor applications, for example, in impedance spectroscopy [1], microelectromechanical systems (MEMS) sensor [4], IoT sensors [5,6], motion detection sensors [7], sensor readout circuit [8], wireless sensor network [9,10,11], basal-body-temperature detection sensor [12], image sensor [13], intelligent human sensing system [14], etc. These voltage controlled oscillators are designed and implemented in CMOS chips, which provides many advantages, for example, low power consumption, compact size, low voltage operation, high speed, etc. However, if the investment costs for fabricating these chips are considered, these CMOS VCOs are worth to be mass-produced. For a specific purpose design or a small circuit quantity, the design of an electronic circuit using commercially available ICs is better. With this feature, it is still cheaper and more convenient compared to the chip fabrication [15]. The synthesis of an analog circuit using commercially available ICs have been continuously proposed [16,17,18,19,20]. Especially the designs of voltage or current controlled quadrature sinusoidal oscillators using commercially available ICs have been found in the open literature [21,22,23,24,25,26,27,28,29,30,31]. Moreover, the linear voltage controlled sinusoidal oscillator using commercially available ICs-based CCII was proposed in [32].

This paper introduces the quadrature sinusoidal oscillator for phase sensitive detection based on commercially available ICs. The frequency of oscillation and the condition of oscillation are electronically and independently controlled. The proposed oscillator consists of the same type of commercially available IC, LT1228 with grounded capacitors. The amplitude of the quadrature waveforms is constant during frequency tuning. Additionally, the amplitude of the quadrature voltage waveform can be controlled. 

## 2. Circuit Description

### 2.1. Concept to Synthesize the Quadrature Sinusoidal Oscillator 

**Figure 2:**
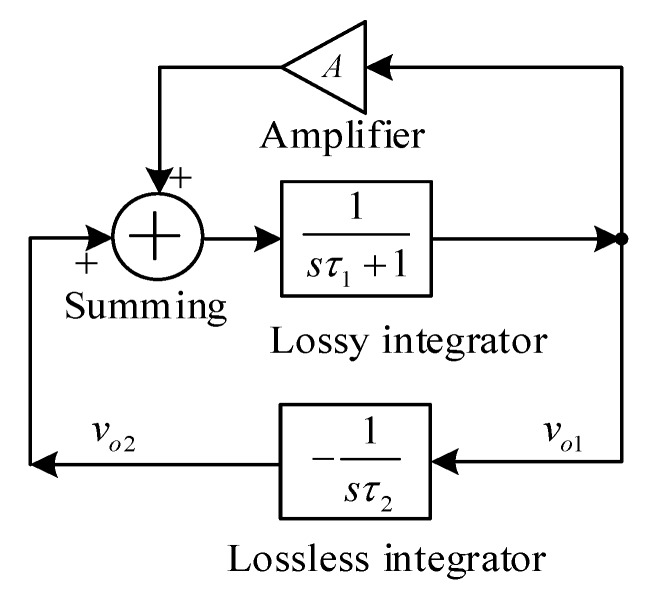
Basic block diagram of quadrature sinusoidal oscillator.

The aim of this research is to synthesis the circuit which can generate the quadrature sinusoidal waveform with an independent control of a generated frequency and condition of oscillation. The quadrature sinusoidal oscillator can be realized by using the basic block given in Figure 2. The sub-circuit consists of a lossy integrator, inverting lossless integrator, amplifier, and summing circuit. All sub-circuits are in the voltage-mode system. The variables τ_1_, τ_2_, and A are the time constant of the lossy integrator, lossless integrator, and the gain of the amplifier, respectively. The quadrature sinusoidal output nodes v_o1_ and v_o2_ are at the input and output of the lossless integrator.

The second order characteristic equation of the basic block in Figure 2 is given by the following expression:

(3) $$s^{2}\tau_{1}\tau_{2}+s\tau_{2}\left(1-A\right)+1=0,$$

where s = jω, the characteristic equation in (3) becomes

(4) $$\left(1-\omega^{2}\tau_{1}\tau_{2}\right)+j\omega \tau_{2}\left(1-A\right)=0.$$

Thus, the frequency of oscillation (f_0_) is obtained by

(5) $$f_{0}=\frac{1}{2\pi }\sqrt{\frac{1}{\tau_{1}\tau_{2}}}.$$

From (5), the linear control of f_0_ is achieved by simultaneously changing τ_1_ and τ_2_ (τ_1_ = τ_2_ = τ). In practice, this control will be more discussed later. Form (4), the condition of oscillation is given by

(6) A≥1.

From (6), the voltage gain A must be slightly higher than one to assure oscillation [1].

The magnitude ratio of the quadrature sinusoidal output voltage v_o2_ and v_o1_ is given by

(7) $${\left|\frac{v_{o2}}{v_{o1}}\right|}_{\tau_{1}=\tau_{2}}=\frac{1}{\omega_{0}\tau_{1}}=1.$$

It is found from (7) that not only the linear control of f_0_ is achieved, the magnitude ratio of two quadrature output voltages is also equal. The phase relationship of v_o2_ and v_o1_ is given by

(8) $$\theta_{\frac{v_{o2}}{v_{o1}}}=90^{{}^{\circ}}.$$

### 2.2. Active Element

**Figure 3:**
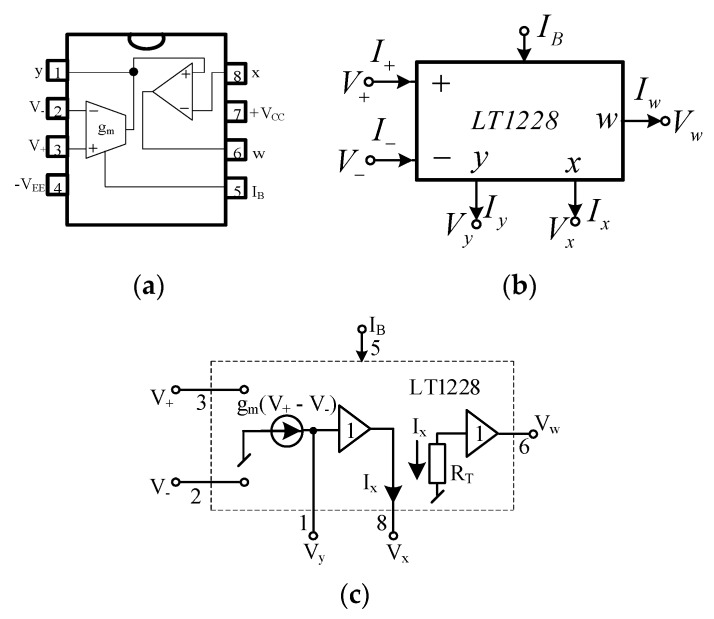
LT1228. (**a**) Pin; (**b)** Symbol; (**c**) Equivalent circuit.

According to (5) and (6), the frequency of oscillation is a function of the time constants τ_1_ and τ_2_, and the condition of oscillation is a function of voltage gain A. Thus, the electronic control of these parameters in the integrators and amplifier can be achieved by using an electronically controllable active element. The LT1228, commercially available IC from Linear Technology [33], is used for this synthesis. This active device combines the very fast transconductance amplifier (OTA) and the current feedback amplifier (CFA) with a wide range of a power supply voltage (±2 V to ±15 V). The transconductance gain (g_m_) of LT1228 is proportional to the DC bias current (I_B_). This IC is packed into an eight-pin dual in-line package as shown in Figure 3a [15]. However, in order to easily draw the electrical scheme, the LT1228 can be illustrated as an electrical symbol in Figure 3b. The equivalent circuit of LT1228 is drawn as shown in Figure 3c. The ideal terminal characteristic of LT1228 is shown in (9):

(9) $$\left(\begin{array}{c} I_{v+}\\ I_{v-}\\ I_{y}\\ \begin{array}{l} V_{x}\\ V_{w} \end{array} \end{array}\right)=\left(\begin{array}{ccccc} 0 & 0 & 0 & 0 & 0\\ 0 & 0 & 0 & 0 & 0\\ g_{m} & -g_{m} & 0 & 0 & 0\\ 0 & 0 & 1 & 0 & 0\\ 0 & 0 & 0 & Z_{T} & 0 \end{array}\right)\left(\begin{array}{c} V_{+}\\ V_{-}\\ V_{y}\\ \begin{array}{l} I_{x}\\ I_{w} \end{array} \end{array}\right).$$

From (9), the Z_T_ represents the trans-resistance gain of the current feedback amplifier. Ideally, the R_T_ approaches infinity. The transconductance gain, g_m_, is controlled by the bias current with the following expression:

(10) $$g_{m}=10I_{B}.$$

### 2.3. Proposed Core Quadrature Sinusoidal Oscillator

**Figure 4:**
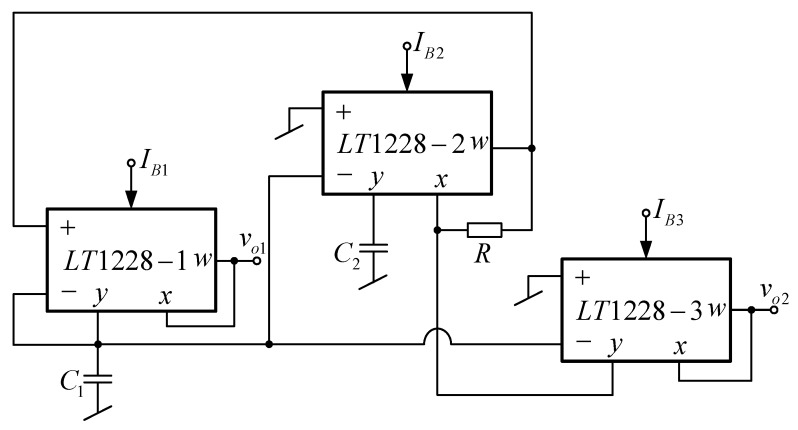
Proposed core quadrature sinusoidal oscillator.

Using the principle presented above, the proposed quadrature oscillator can be synthesized as shown in Figure 4. The proposed oscillator consists of three LT1228s, two grounded capacitors (C_1_ and C_2_) and one resistor R, where the lossy integrator is constructed from LT1228-1 and C_1_, the lossless integrator is constructed from LT1228-2 and C_2_, and the voltage amplifier with a voltage summing circuit is constructed from LT1228-3, R, and LT1228-2. Note that most of commercially available IC based quadrature oscillators proposed in [22,23,24,25,26,27,28,29,30,31] use the difference type of commercially available ICs, but the proposed oscillator uses only three LT1228s. Only the quadrature oscillator in [21] uses the same type of commercially available IC (LT1228), but it requires five LT1228s. The time constants for the first and second integrator are respectively τ_1_ = C_1_/g_m1_ and τ_2_ = C_2_/g_m2_, while the voltage gain of the amplifier is A = g_m3_R. The DC bias currents I_B1_, I_B2_, and I_B3_ are used to control the g_m1_, g_m2_, and g_m3_, respectively. The quadrature output voltage nodes v_o1_ and v_o2_ are of low impedance, which can connect to the impedance sensor or another circuit in the phase sensitive detection system without the need of buffer devices. The characteristic equation of the oscillator in Figure 4 is given by the following equation:

(11) $$s^{2}C_{1}C_{2}+sC_{2}g_{m1}\left(1-g_{m3}R\right)+g_{m1}g_{m2}=0$$

Thus, the frequency of oscillation (f_0_) is obtained by

(12) $$f_{0}=\frac{1}{2\pi }\sqrt{\frac{g_{m1}g_{m2}}{C_{1}C_{2}}}$$

and the condition of oscillation is given by

(13) $$g_{m3}R\geq 1$$

Substituting (10) into (12) and (13), the frequency of oscillation, which is independently and electronically controlled from the condition of oscillation, is given by

(14) $$f_{0}=\frac{5}{\pi }\sqrt{\frac{I_{B1}I_{B2}}{C_{1}C_{2}}}$$

and the condition of oscillation, which is independently and electronically controlled from the frequency, is given by

(15) $$I_{B3}\geq \frac{1}{10R}$$

From (15), the bias current I_B3_ must be slightly higher than 1/10R to assure oscillation [1]. From (14), the linear and electronic control of f_0_ is achieved by simultaneously changing I_B1_ and I_B2_ (I_B1_ = I_B2_ = I_B_). Then, the frequency of oscillation becomes

(16) $$f_{0}=\frac{5I_{B}}{\pi \sqrt{C_{1}C_{2}}}$$

The similar feature for electronic control of FO and CO is available in the presented quadrature oscillators in [21,22,23,24,25,26,28,29,30]. However, the option of a linear tune of FO is not obtained for works in Refs. [23,30,31]. Moreover, both frequency and condition of oscillation of the circuits in [27,31] are not electronically controlled. The voltage transfer of the quadrature sinusoidal output voltage v_o2_ and v_o1_ is given by

(17) $$\frac{v_{o2}}{v_{o1}}=-\frac{g_{m2}}{sC_{2}}=-\frac{10I_{B2}}{sC_{2}}$$

From (17), the phase difference of v_o2_ and v_o1_ is given by

(18) $$\theta_{\frac{v_{o2}}{v_{o1}}}=90^{{}^{\circ}}$$

From the deliberations stated above, the tuning of the frequency of oscillation is done by simultaneously changing I_B1_ and I_B2_ (I_B1_ = I_B2_ = I_B_) and setting C_1_ = C_2_, the magnitude ration of the quadrature sinusoidal output voltage v_o2_ and v_o1_ is

(19) $$\left|\frac{v_{o2}}{v_{o1}}\right|=1$$

From (19), the magnitude ratio of the two quadrature output voltages is equal along the frequency tuning range.

### 2.4. Proposed Quadrature Sinusoidal Oscillator with Amplitude Controllability

**Figure 5:**
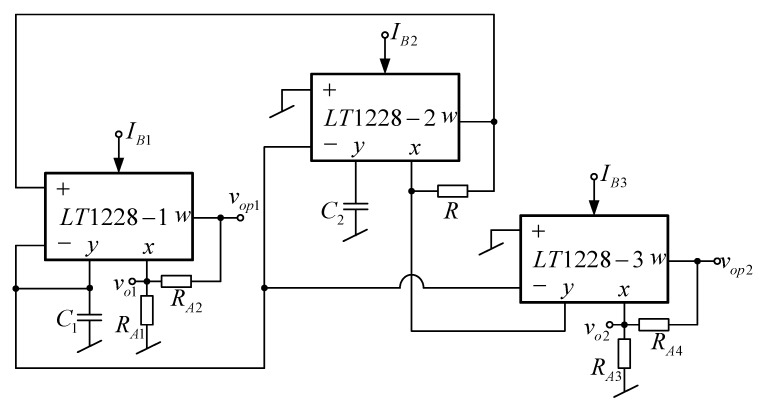
Proposed quadrature sinusoidal oscillator with gain controllability of quadrature output.

The amplitude of the output voltage v_o1_ can be controllable by adding the resistors R_A1_ and R_A2_ as shown in Figure 5. This amplitude controllable output voltage is defined as v_op1_. The voltage gain of the first output voltage is

(20) $$A_{V1}=\frac{v_{op1}}{v_{o1}}=\left(\frac{R_{A2}}{R_{A1}}+1\right)$$

The amplitude of the output voltage v_o2_ can be controllable by adding the resistors R_A2_ and R_A3_ as shown in Figure 5. This amplitude controllable output voltage is defined as v_op2_. The voltage gain of the second output voltage is

(21) $$A_{V2}=\frac{v_{op2}}{v_{o2}}=\left(\frac{R_{A4}}{R_{A3}}+1\right)$$

From (20) and (21), it is found that the amplitude of quadrature output voltages is controllable without using additional active devices, but this feature of the commercially available IC based quadrature oscillators in [22,23,24,25,26,27,28,29,30,31] is not available. Furthermore, the quadrature output voltage nodes of the oscillator in [22,23,24,25,27,29,31] are not of low impedance, requiring the voltage buffer for cascading. 

### 2.5. Practical Implementation for Voltage Controlled Oscillator

**Figure 6:**
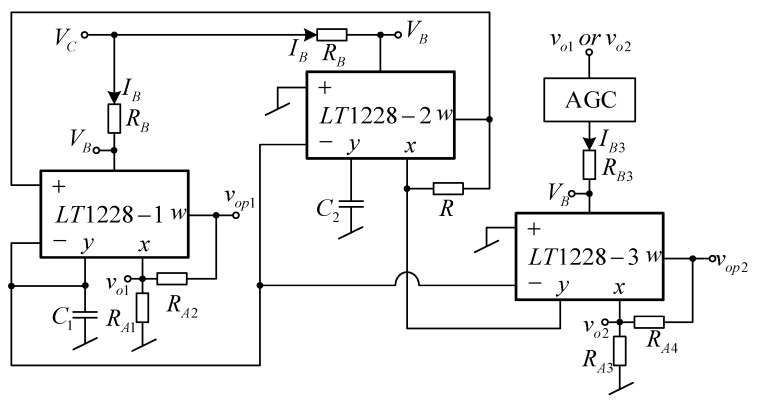
Proposed quadrature sinusoidal oscillator with voltage control and automatic gain control (AGC).

From (16), the linear tuning of frequency can be done by simultaneously adjusting I_B1_ and I_B2_. With this feature, the voltage controllability of the frequency is practically implemented by using the same value of bias resistor R_B_ for LT1228-1 and LT1228-2, then applying the control voltage V_C_ to bias resistor R_B_ as shown in Figure 6. If all LT1228s are biased with the same voltage supplies, the bias voltage V_B_ at pin 5 for each LT1228 is also the same. If the bias voltage V_B_ is two diode voltage drops (V_d_ ≅ 0.7 V) above the negative voltage supply [33], then the value of V_B_ at pin 5 for each LT1228 is given by the following expression

(22) $$V_{B}≅-\left|V_{EE}\right|+1.4$$

where V_EE_ is the negative voltage supply. It is found from (22) that the V_B_ is negative, which makes bias current flowing into pin 5 of LT1228 with the following expression

(23) $$I_{B}=\frac{V_{C}+\left|V_{EE}\right|-1.4}{R_{B}}$$

Substituting (23) into (16), the frequency of oscillation is linearly controlled by control voltage V_C_ with the following expression:

(24) $$f_{0}=\frac{5}{\pi R_{B}\sqrt{C_{1}C_{2}}}\left(V_{C}+\left|V_{EE}\right|-1.4\right)$$

From (24), the frequency of oscillation is easily controlled by a microcomputer or microcontroller using an analog pin or digital-to-analog (DAC) circuit. For amplitude stabilization and automatic control of condition of oscillation, the automatic gain control (AGC) circuit is required [28] as shown in Figure 6. The simplified AGC topology can be found in [28].

### 2.6. Effect of Parasitic Elements

The proposed quadrature sinusoidal oscillator is implemented for typical impedance sensor operation where the frequency range is up to hundreds of Kilo Hertz (kHz) [1]. Therefore, the effect of parasitic elements at high frequency is neglected. These parasitic elements include Z_T_, Z_x_, and Z_w_, where the Z_T_ is the parallel of the parasitic R_T_ and C_T_, the Z_x_ is the series of the parasitic R_x_ and L_x_ appearing at the x terminal, and Z_w_ is the series of parasitic R_w_ and L_w_ appearing at the w terminal. The most significant effects are from the parallel of the parasitic R_+_ and C_+_ at the V_+_ terminal, the parallel of the parasitic R_–_ and C_–_ at V_–_ terminal, and the parallel of the parasitic R_y_ and C_y_ at the y terminal. Taking these parasitic elements into account, the characteristic equation of the core oscillator in Figure 4 is given by the following equation:

(25) $$\left\{\begin{array}{l} s^{2}C_{1}^{*}C_{2}^{*}+s\left(C_{1}^{*}G_{y2}+C_{2}^{*}G_{1}^{*}+C_{2}^{*}g_{m1}-C_{2}^{*}g_{m1}g_{m3}R+C_{y3}g_{m1}g_{m2}R\right)+\\ G_{y2}g_{m1}\left(1-g_{m3}R\right)+G_{y3}g_{m1}g_{m2}R+g_{m1}g_{m2} \end{array}\right\}=0$$

where $C_{1}^{*}=C_{1}+C_{y1}+C_{-1}+C_{-2}+C_{-3}$, $G_{1}^{*}=G_{y1}+G_{-1}+G_{-2}+G_{-3}$, and $C_{2}^{*}=C_{2}+C_{y2}$. Thus, the frequency of oscillation with parasitic effect is obtained by

(26) $$\omega_{0}^{*}=\sqrt{\frac{G_{y2}g_{m1}\left(1-g_{m3}R\right)+G_{v1+}g_{m1}g_{m2}R+g_{m1}g_{m2}}{C_{1}^{*}C_{2}^{*}}}$$

and the condition of oscillation with a parasitic effect is given by

(27) $$\frac{C_{1}^{*}G_{y2}}{C_{2}^{*}g_{m1}}+\frac{G_{1}^{*}}{g_{m1}}+\frac{C_{v1+}g_{m2}R}{C_{2}^{*}}+1\leq g_{m3}R$$

The voltage transfer of the quadrature sinusoidal output voltage v_o2_ and v_o1_ with a parasitic effect is given by

(28) $$\frac{v_{o2}}{v_{o1}}=-\frac{g_{m2}}{sC_{2}^{*}+G_{y2}}$$

From (28), the phase difference of v_o2_ and v_o1_ with a parasitic effect is given by

(29) $$\theta^{*}{}_{\frac{v_{o2}}{v_{o1}}}=180-\tan^{-1}\left(\frac{\omega_{0}^{*}C_{2}^{*}}{G_{y2}}\right)$$

## 3. Results

**Figure 7:**
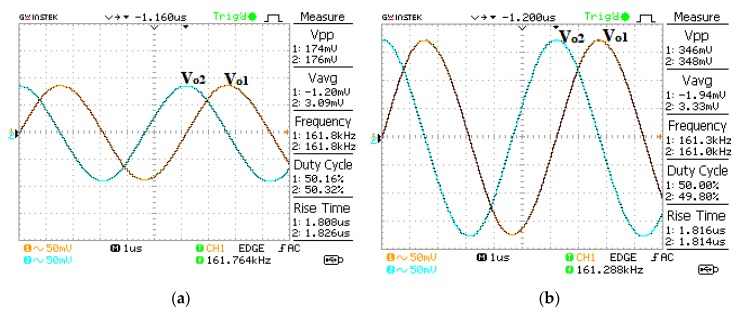
Measured sinusoidal output waveform (**a**) v_o1_ and v_o2_, and (**b**) v_op1_ and v_op2_.

**Figure 8:**
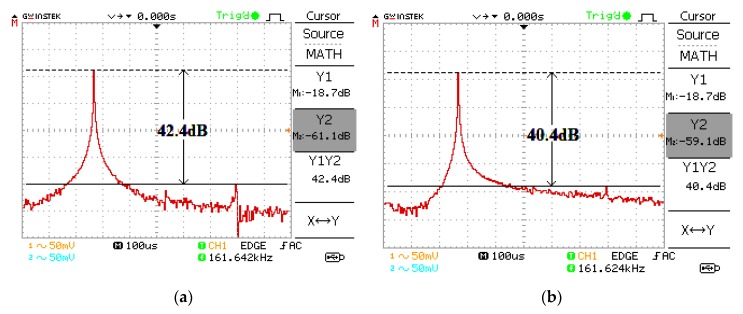
Measured spectrum for (**a**) v_o1_ and (**b**) v_o2_.

**Figure 9:**
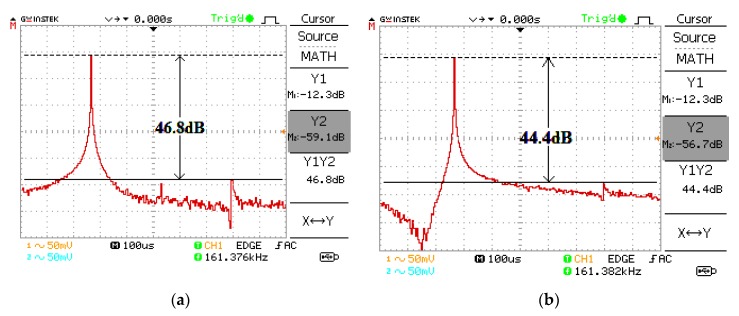
Measured spectrum for (**a**) v_op1_ and (**b**) v_op2_.

The proposed oscillator in Figure 6 is supplied with ±5 V using the GW Instek GPS-3303 power supply. Firstly, the bias currents are set to I_B1_ = I_B2_ = 100 µA and I_B3_ = 103.26 µA. These bias currents are controlled by the bias resistor, R_B_. The DC bias currents are measured with a Fluke 289 multimeter. The resistor R (setting the condition of oscillation) is 1 kΩ and the resistors R_A1_, R_A2_, R_A3_, and R_A4_ (setting the amplitude of two quadrature sinusoidal waveforms) are 1 kΩ. The capacitors C_1_ and C_2_ are chosen as 1 nF. The measured quadrature sinusoidal output waveforms with the GW Instek GPS-1072-U oscilloscope for v_o1_, v_o2_, v_op1_, and v_op2_ are shown in Figure 7. The theoretical frequency of oscillation calculating from (16) yields 159.23 kHz, while the experimental frequency of oscillation is 161.76 kHz. The deviation of frequency of oscillation from the theory and experiment is about 1.58%, which is stemmed from the parasitic element effect as the analysis in Section 2.6. The theoretical voltage gains A_V1_ and A_V2_ in (20) and (21) are 2, while the experimental A_V1_ and A_V2_ are 1.98 and 1.97, respectively. Figure 8 shows the v_o1_ and v_o2_ spectrum analysis, which demonstrates that the total harmonic distortion (THD) of v_o1_ and v_o2_ are −42.4 dB (0.759%) and −40.4 dB (0.955%). Figure 9 shows the v_op1_ and v_op2_ spectrum analysis, which demonstrates that the THDs of v_op1_ and v_op2_ are −46.8 dB (0.457%) and −44.4 dB (0.603%), respectively. The power consumption is approximately 198.5 mW. 

**Figure 10:**
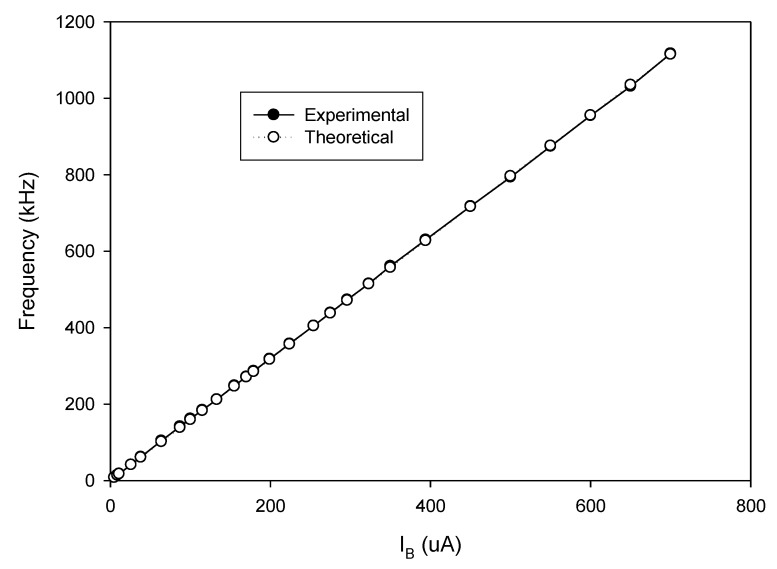
The plot of the frequency of oscillation vs. I_B_ (I_B1_ = I_B2_ = I_B_).

**Figure 11:**
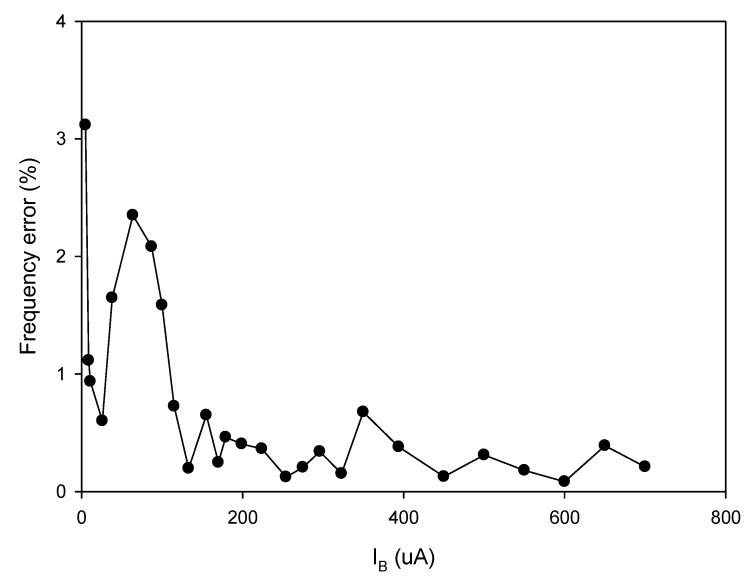
The plot of the frequency error vs. I_B_ (I_B1_ = I_B2_ = I_B_).

**Figure 12:**
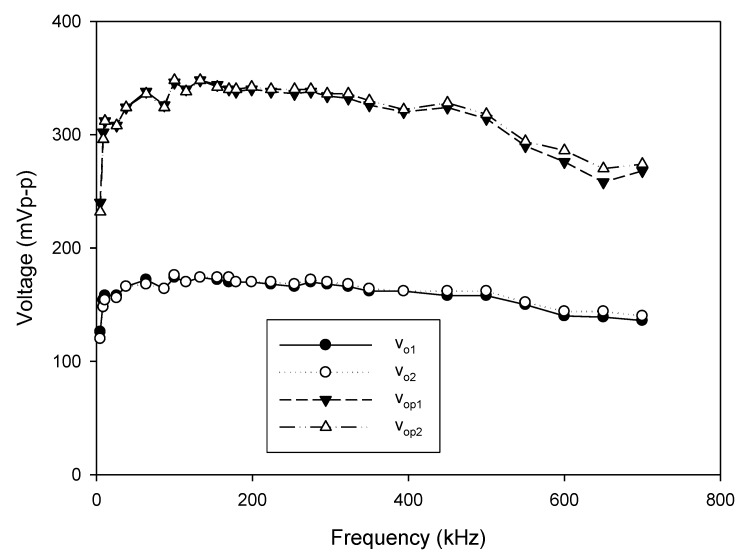
Measurement of the peak-to-peak voltage of quadrature sinusoidal waveforms.

**Figure 13:**
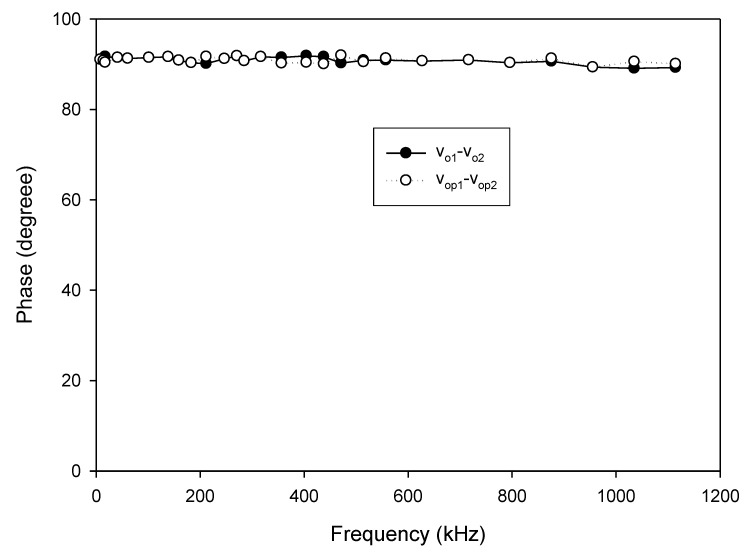
Phase difference of the quadrature output voltage.

**Figure 14:**
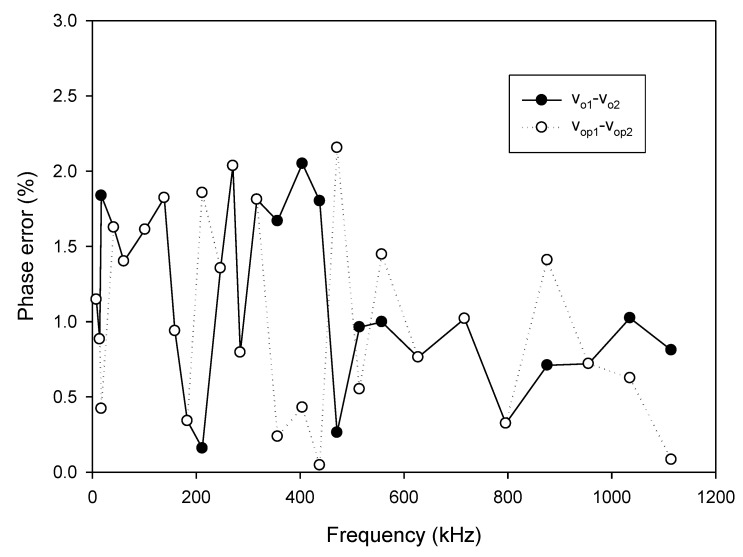
The plot of the phase error vs. frequency of oscillation (FO).

**Figure 15:**
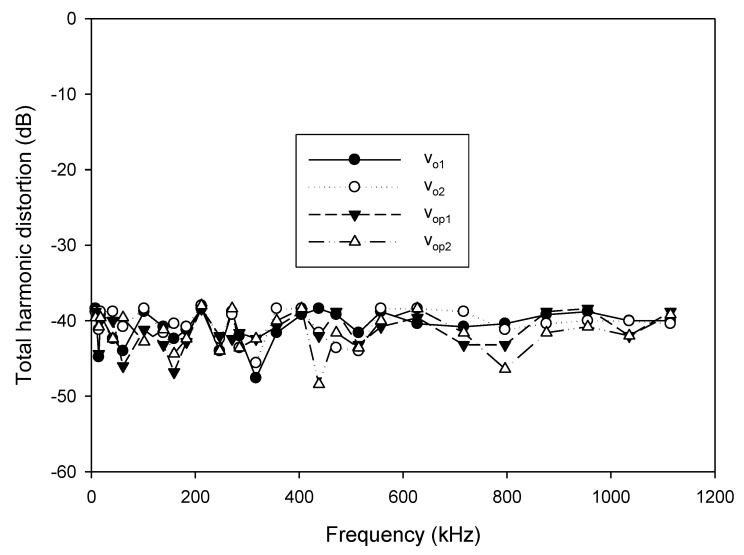
Total harmonic distortion of the quadrature output voltage waveforms.

**Figure 16:**
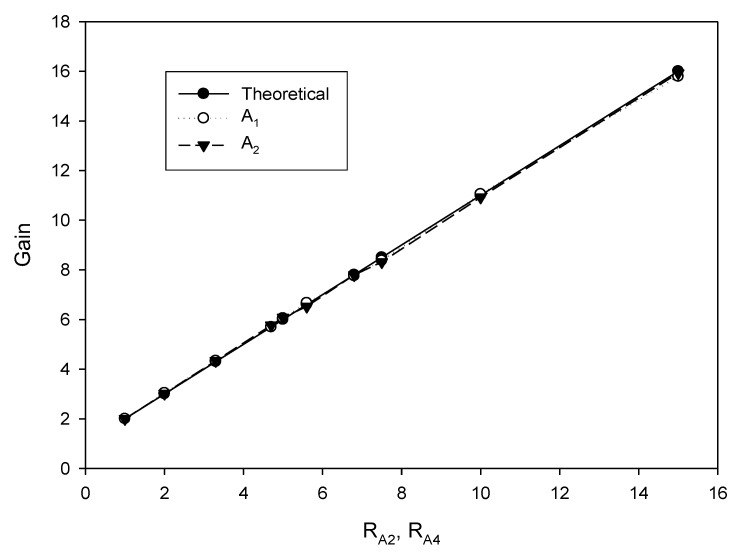
The voltage gains as a function of the resistors R_A2_ and R_A4_.

As analyzed in (16), the linear tune of the frequency can be achieved by simultaneously adjusting the bias currents I_B1_ and I_B2_ (I_B1_ = I_B2_ = I_B_). The frequency of oscillation as a function of the bias current is shown in Figure 10. In this experiment, the bias current I_B_ is sweep swept from 5 µA to 700 µA. The experimental frequency varies from 8.21 kHz to 1117.51 kHz. With this frequency range, the proposed circuit is sufficient to be used in the typical impedance sensor application [1]. The plot of the frequency error vs I_B_ (I_B1_ = I_B2_ = I_B_) is shown in Figure 11. The frequency error stems from the parasitic resistances and capacitances as analyzed in (26). The percentages of relative error swing from 0.084% (I_B_ = 600 µA) at a frequency of 954.61 kHz to 3.12% (I_B_ = 5 µA) at a frequency of 8.21 kHz with an absolute mean error lower than 0.728%. It is found that the maximum frequency error occurred at the low value of the bias current. To reduce this error, the bias current (I_B_) should be up to 100 µA. However, if the low frequency of oscillation with a high value of bias current is required, the capacitance of C_1_ and C_2_ should be practically set to a high value as analyzed in (16). Note that the linearly and electronically controllable quadrature oscillators [21,22,24,25,26,27,28,29] using commercially available ICs can operate at the frequency up to a MHz range, but the result about frequency error throughout the frequency range does not show. Figure 12 shows the measurement of the peak-to-peak voltage of quadrature sinusoidal waveforms. It is found from the result in Figure 12 that the amplitudes of couple output voltage v_o1_–v_o2_ and v_op1_–v_op2_ are quite the same when the frequency is tuned as analyzed in (19). The phase difference of the quadrature sinusoidal voltage waveform v_o1_–v_o2_ and v_op1_–v_op2_ is depicted in Figure 13. The percent of phase error against the frequency of oscillation is plotted in Figure 14. The relative phase error of v_o1_–v_o2_ is lower than 2.05% with an absolute mean error lower than 1.15%, while the relative phase error of v_op1_–v_op2_ is lower than 2.16% with an absolute mean error lower than 1.04%. The phase error stems from the parasitic resistance, R_y2_ and capacitance, C_y2_ as analyzed in (29). It should be noted that the phase error of the proposed oscillator is quite closed to oscillators in Refs. [22,23,24,26,27,28]. However, the quadrature phase error of the oscillator in Ref. [25] is quite higher (2.22–43.33%) than the proposed oscillator. The total harmonic distortion of the quadrature output voltage waveforms is shown in Figure 15. It is found that the total harmonic distortion is lower than −34.8 dB. As analyzed in (20) and (21), the amplitude of the quadrature output voltage v_op1_ and v_op2_ can be adjusted via R_A1_, R_A2_, R_A3_, and R_A4_. The voltage gains as a function of the resistors R_A2_ and R_A4_ are shown in Figure 16. In this experiment, the value of R_A2_ and R_A4_ is swept from 1 kΩ to 15 kΩ. With this tuning, the voltage gains vary from 1.97 to 15.92.

## 4. Comparison

A comparison of a proposed oscillator with the previous voltage or current controlled quadrature sinusoidal oscillators using commercially available ICs published in the open literature [21,22,23,24,25,26,27,28,29,30,31] is given in Table 1. In this comparison, the performances of the proposed oscillator were tested with ±3 V and ±5 V power supplies. It is found that most oscillators [22,23,24,25,26,27,28,29,30,31] use the difference type of commercially available ICs, but the proposed oscillator uses only three LT1228s. Only the quadrature oscillator in [21] uses the same type of commercially available IC (LT1228), but it requires five LT1228s. Both frequency and condition of oscillation of the circuits in [27,31] are not electronically controlled. The quadrature output voltage nodes of the oscillator in [22,23,24,25,27,29,31] are not of low impedance and require the voltage buffer for cascading. The quadrature output amplitudes are not controllable [21,22,23,24,25,26,27,28,29,30,31] and need additional voltage amplifier circuits for adjusting the quadrature output waveforms. The frequency of oscillation in [23,30,31] is not linearly tuned. Most of quadrature oscillators are tested at ±5 V power supplies (except Ref. 21), but the proposed oscillator can operate at lower power supplies (±3 V). However, the performances of the proposed oscillator with a wider frequency range, lower frequency error, lower phase error, and lower THD are achieved when the circuits are supplied with ±5 V as shown in Table 1. The oscillators [22,23,24,25,26,27,28,29,30,31] are designed to operate at the frequency of up to a MHz range, the operating frequency of the proposed oscillator can be also varied from 8.21 kHz to 1.11 MHz, which is sufficient to be used in the typical impedance sensor application [1]. The frequency error for all quadrature oscillators [21,22,23,24,25,26,27,28,29,30,31] is not available or has not been tested. The phase error and THD of the proposed oscillator are quite closed to other oscillators. 

## 5. Conclusions

A new commercially available IC based current/voltage controlled quadrature sinusoidal oscillators for a phase sensitive detection (PSD) application is presented in this paper. The core oscillator is constructed from three LT1228s, two grounded capacitors and one resistor. The quadrature output voltage nodes are of low impedance, which can connect to other circuits without the use of the external voltage buffers. The frequency of oscillation is electronically and linearly tuned from 8.21 kHz to 1117.51 kHz without affecting the condition of oscillation. In addition, the condition of oscillation is electronically controlled. By adding the bias resistors R_B_, the frequency and condition of the proposed oscillator can be adjusted by a controlled voltage. Moreover, the amplitude of the quadrature output voltage waveform can be adjusted by adding the resistors R_A1_, R_A2_, R_A3_, and R_A4_ without the requirement of additional active devices. The amplitude of a quadrature output is equal for all frequency ranges. The measurement results demonstrate that the proposed oscillator works in a wide frequency range and it is a suitable choice for an instrument-off-the-shelf device. 

## Figures and Tables

**Table 1:** Comparison of relevant voltage or current controlled quadrature oscillators using commercially available ICs.

Ref.	Number of Commercial Chips	Electronic Tune Both FO and CO	Low Output Impedance	Amplitude Controllability	FO Tuning	V_CC_	FO Range (MHz)	FO Error Range (%)	Phase Error (%)	THD (%)
[21]	**5** (LT1228)	Yes	Yes	No	Linear	±15 V	N/A	N/A	N/A	N/A
[22]	**5** (3 AD835, 2 OPA615)	Yes	No	No	Linear	±5 V	2.69–30.18	N/A	<5.56	<3
[23]	**6** (1 AD835, 1 AD830, 4 EL2082)	Yes	No	No	Quadratic	±5 V	0.05–4.00	N/A	<2.23	<1
[24]	**5** (2 VCA810, 3 EL2082)	Yes	No	No	Linear	±5 V	0.153–4.94	N/A	<1.12	<1.7
[25]	**4** (1 AD830, 1 AD835, 2 EL2082)	Yes	No	No	Linear	±5 V	1.82–20.18	N/A	2.22–43.33	<1.2
[26]	**5** (3 EL2082, 2 AD8138)	Yes	Yes	No	Linear	±5 V	0.05–6.82	N/A	1.11–2.22	<0.9
[27]	**3** (2 OPA660, EL2082)	No	No	No	Linear	±5 V	0.50–7.91	N/A	<3	<0.5
[28]	**6** (2 EL2082, 1 AD8138, 1 VCA810, 2 BUF634)	Yes	Yes	No	Linear	±5 V	0.25–8.00	N/A	<2.81	<8.0
[29]	**4** (3 EL2082, 1 OPA860)	Yes	No	No	Linear	±5 V	0.31–7.87	N/A	N/A	<0.90
[30]	**3** (2 EL2082, 1 OPA860)	Yes	Yes	No	Square root	±5 V	0.20–1.30	N/A	N/A	<1.5
[31]	**3** (1 EL2082, 2 OPA860)	No	No	No	Square root	±5 V	0.20–1.20	N/A	N/A	<4.0
This research	**3** (LT1228)	Yes	Yes	Yes	Linear	±3 V	0.15–0.65	3.61–8.81	<2.96	<1.82
						±5V	0.08–1.11	0.08–3.12	<1.15	<1.26

N/A—not available or not tested. Amplitude of the quadrature output waveform in [27,30,31] is not equal during FO tuning.

## References

[1] Márquez A., Pérez-Bailón J., Calvo B., Medrano N., Martínez P.A. (2018). A CMOS self-contained quadrature signal generator for soc impedance spectroscopy. Sensors.

[2] Arshad A., Khan S., Zahirul Alam A., Tasnim R. Analysis of phase detection circuit for human activity. Proceedings of the IEEE International Conference on Smart Instrumentation, Measurement and Applications.

[3] Blair D.P., Sydenham P.H. (1975). Phase sensitive detection as a means to recover signals buried in noise. J. Phys. E Sci. Instrum..

[4] Quintero A., Cardes F., Perez C., Buffa C., Wiesbauer A., Hernandez L. (2019). A VCO-based CMOS readout circuit for capacitive MEMS microphones. Sensors.

[5] Kim S.J., Kim D.G., Oh S.J., Lee D.S., Pu Y.G., Hwang K.C., Yang Y., Lee K.Y. (2019). A Fully Integrated Bluetooth Low-Energy Transceiver with Integrated Single Pole Double Throw and Power Management Unit for IoT Sensors. Sensors.

[6] Wang S.-F., Chang Y.-W., Tang C.-Y. (2018). A novel dual-band six-phase voltage-control oscillator. Sensors.

[7] Ha K.-W., Lee J.-Y., Kim J.-G., Baek D. (2018). Design of dual-mode local oscillators using CMOS technology for motion detection sensors. Sensors.

[8] Cardes F., Quintero A., Gutierrez E., Buffa C., Wiesbauer A., Hernandez L. (2018). SNDR limits of oscillator-based sensor readout circuits. Sensors.

[9] Liu D., Hu A., Zhang K. (2018). A quadrature single side-band mixer with passive negative resistance in software-defined frequency synthesizer. Sensors.

[10] Liang Z., Li B., Huang M., Zheng Y., Ye H., Xu K., Deng F. (2017). A low cost bluetooth low energy transceiver for wireless sensor network applications with a front-end receiver-matching network-reusing power amplifier load inductor. Sensors.

[11] El-Desouki M.M., Qasim S.M., BenSaleh M., Deen M.J. (2013). Single-chip fully integrated direct-modulation CMOS RF transmitters for short-range wireless applications. Sensors.

[12] Yang C.-L., Zheng G.-T. (2015). Wireless low-power integrated basal-body-temperature detection systems using teeth antennas in the MedRadio band. Sensors.

[13] Cao Y., Pan X., Zhao X., Wu H. (2014). An analog gamma correction scheme for high dynamic range CMOS logarithmic image sensors. Sensors.

[14] Kim J.-W., Takao H., Sawada K., Ishida M. (2007). Integrated inductors for RF transmitters in CMOS/MEMS smart microsensor systems. Sensors.

[15] Siripongdee S., Jaikla W. (2017). Electronically controllable grounded inductance simulators using single commercially available IC: LT1228. AEU-Int. Electron. Commun..

[16] Yuce E., Verma R., Pandey N., Minaei S. (2019). New CFOA-based first-order all-pass filters and their applications. AEU-Int. Electron. Commun..

[17] Barile G., Safari L., Ferri G., Stornelli V. (2019). A VCII-based stray insensitive analog interface for differential capacitance sensors. Sensors.

[18] Han B., Xu Y., Dong F. (2017). Design of current source for multi-frequency simultaneous electrical impedance tomography. Rev. Sci. Instrum..

[19] Yuce E. (2017). DO-CCII/DO-DVCC based electronically fine tunable quadrature oscillators. J. Circuit. Syst. Comput..

[20] Yuce E., Minaei S., Cicekoglu O. (2006). Limitations of the simulated inductors based on a single current conveyor. IEEE Trans. Circuits Syst..

[21] Wang S.-F., Chen H.-P., Ku Y., Lin Y.-C. (2019). Versatile tunable voltage-mode biquadratic filter and its application in quadrature oscillator. Sensors.

[22] Sotner R., Polak L., Petrzela J., Langhammer L. (2018). Practical design of the voltage controllable quadrature oscillator for operation in MHz bands employing new behavioral model of variable-voltage-gain current conveyor of second generation. J. Comput. Electron..

[23] Sotner R., Jerabek J., Herencsar N., Petrzela J. (2018). Methods for Extended Tunability in Quadrature Oscillators Based on Enhanced Electronic Control of Time Constants. IEEE Trans. Instrum. Meas..

[24] Sotner R., Herencsar N., Jerabek J., Langhammer L., Polak J. (2017). On practical construction of electronically controllable compact current amplifier based on commercially available elements and its application. AEU-Int. Electron. Commun..

[25] Sotner R., Jerabek J., Langhammer L., Polak J., Herencsar N., Prokop R., Petrzela J., Jaikla W. (2015). Comparison of Two Solutions of Quadrature Oscillators With Linear Control of Frequency of Oscillation Employing Modern Commercially Available Devices. Circuits Syst. Signal Process..

[26] Sotner R., Herencsar N., Jerabek J., Jerabek J., Koton J., Dostal T., Vrba K. (2014). Electronically controlled oscillator with linear frequency adjusting for four-phase or differential quadrature output signal generation. Int. J. Circuit Theory Appl..

[27] Sotner R., Jerabek J., Herencsar N., Petrzela J., Vrba K., Kincl Z. (2014). Linearly tunable quadrature oscillator derived from LC Colpitts structure using voltage differencing transconductance amplifier and adjustable current amplifier. Analog Integr. Circuits Signal Process..

[28] Sotner R., Hrubos Z., Herencsar N., Jerabek J., Dostal T., Vrba K. (2014). Precise electronically adjustable oscillator suitable for quadrature signal generation employing active elements with current and voltage gain control. Circuits Syst. Signal Process..

[29] Sotner R., Lahiri A., Kartci A., Herencsar N., Jerabek J., Vrba K. (2013). Design of novel precise quadrature oscillators employing ECCIIS with electronic control. Adv. Electr. Comput. Eng..

[30] Šotner R., Hruboš Z., Ševčík B., Slezák J., Petržela J., Dostál T. (2011). An example of easy synthesis of active filter and oscillator using signal flowgraph modification and controllable current conveyors. J. Electr. Eng..

[31] Sotner R., Jerabek J., Prokop R., Vrba K. (2011). Current gain controlled CCTA and its application in quadrature oscillator and direct frequency modulator. Radioengineering.

[32] Sotner R., Jerabek J., Langhammer L., Dvorak J. (2019). Design and analysis of CCII-based oscillator with amplitude stabilization employing optocouplers for linear voltage control of the output frequency. Electronics.

[33] Linear Technology, LT1228: 100 MHz Current Feedback Amplifier with DC Gain Control. https://www.analog.com/media/en/technical-documentation/data-sheets/1228fd.pdf.

